# Species abundance, composition, and nocturnal activity of female *Anopheles* (Diptera: Culicidae) in malaria-endemic villages of Papua New Guinea: assessment with barrier screen sampling

**DOI:** 10.1186/s12936-019-2742-x

**Published:** 2019-03-25

**Authors:** John B. Keven, Michelle Katusele, Rebecca Vinit, Gussy Koimbu, Naomi Vincent, Edward K. Thomsen, Stephan Karl, Lisa J. Reimer, Edward D. Walker

**Affiliations:** 1 Vector Borne Diseases Unit, Papua New Guinea Institute of Medical Research, Madang, Papua New Guinea; 2 Department of Microbiology and Molecular Genetics, Michigan State University, East Lansing, MI USA; 3 Liverpool School of Tropical Medicine, Liverpool, UK; 4 Walter and Eliza Hall Institute of Medical Research, Parkville, VIC Australia; 5 Department of Medical Biology, Melbourne University, Parkville, VIC Australia

**Keywords:** *Anopheles*, Barrier, Blood-fed, Bush, Mosquitoes, Screen, Unfed, Village

## Abstract

**Background:**

Community composition of *Anopheles* mosquitoes, and their host-seeking and peridomestic behaviour, are important factors affecting malaria transmission. In this study, barrier screen sampling was used to investigate species composition, abundance, and nocturnal activity of *Anopheles* populations in villages of Papua New Guinea.

**Methods:**

Mosquitoes were sampled from 6 pm to 6 am in five villages from 2012 to 2016. The barrier screens were positioned between the village houses and the perimeter of villages where cultivated and wild vegetation (“the bush”) grew thickly. Female *Anopheles* that rested on either village or bush side of the barrier screens, as they commuted into and out of the villages, were captured. Similarity in species composition among villages was assessed. Mosquitoes captured on village and bush sides of the barrier screens were sorted by feeding status and by hour of collection, and their numbers were compared using negative binomial generalized linear models.

**Results:**

Females of seven *Anopheles* species were present in the sample. Species richness ranged from four to six species per village, but relative abundance was highly uneven within and between villages, and community composition was similar for two pairs of villages and highly dissimilar in a fifth. For most *Anopheles* populations, more unfed than blood-fed mosquitoes were collected from the barrier screens. More blood-fed mosquitoes were found on the side of the barrier screens facing the village and relatively more unfed ones on the bush side, suggesting commuting behaviour of unfed host-seeking females into the villages from nearby bush and commuting of blood-fed females away from villages towards the bush. For most populations, the majority of host-seeking mosquitoes arrived in the village before midnight when people were active and unprotected from the mosquitoes by bed nets.

**Conclusion:**

The uneven distribution of *Anopheles* species among villages, with each site dominated by different species, even among nearby villages, emphasizes the importance of vector heterogeneity in local malaria transmission and control. Yet, for most species, nocturnal activity patterns of village entry and host seeking predominantly occurred before midnight indicating common behaviours across species and populations relative to human risk of exposure to *Anopheles* bites.

**Electronic supplementary material:**

The online version of this article (10.1186/s12936-019-2742-x) contains supplementary material, which is available to authorized users.

## Background

Sampling adult female *Anopheles* mosquitoes is crucial for studies of bionomics, to estimate population parameters, quantify malaria and filarial parasite transmission, and evaluate vector-targeted disease interventions [1–3]. Three common sampling methods are (i) the human landing catch (HLC), (ii) baited or unbaited light trap (LT), and (iii) resting collection (RC). These can be conducted in various locations such as within human or animal domiciles or amongst vegetation. The HLC method involves human volunteers luring and capturing host-seeking mosquitoes as they land on exposed legs [3]. The method provides direct estimates of the human-biting rate (HBR) and infectious biting rate (IBR), and provides a means for characterizing such important bionomic properties as nocturnal periodicity of the biting cycle [2, 3]. The LT method involves the use of battery-powered suction devices fitted with light bulbs and/or artificial host odors to attract and trap host-seeking adult mosquitoes. The method provides estimates of relative mosquito density, indirect estimates of IBR, and can be used to assess species diversity, community composition, relative abundance, and distribution [1, 4–9]. The RC method involves search and capture of endophilic mosquitoes settled inside human houses or animal sheds, and exophilic mosquitoes resting in the surrounding vegetation or in intentionally placed resting shelters [1, 3, 10]. It may involve spraying insecticides that rapidly knock down mosquitoes in indoor spaces [10]. Such collections are used to study mosquito resting habits, recover blood-fed mosquitoes for analysis of host selection, to analyse distribution in space and time, and to evaluate the effect of residual insecticide treatments on endophilic vectors [3, 11].

These mosquito sampling methods have been applied in Papua New Guinea (PNG)—a country where malaria is endemic with all four solely-human malaria species present and where the IBR can exceed 1000 infectious bites per person per year in some locations [12, 13]. Over 20 different species of *Anopheles* are found in PNG [5, 14, 15], of which 11 have been incriminated as vectors of human malaria [14]. Seven of these species, namely *Anopheles farauti* sensu stricto (*s.s*.), *Anopheles hinesorum*, *Anopheles farauti* no. 4, *Anopheles punctulatus s.s*., *Anopheles koliensis*, *Anopheles longirostris* and *Anopheles bancroftii*, are vectors of malaria in lowland areas of PNG, including Madang province, where malaria is highly endemic [13, 16–18]. The first five are major vectors whereas the last two play a minor role in the transmission of malaria in PNG [13, 14, 16, 17, 19–21]. Various aspects of these vector species including HBR, IBR, nocturnal biting cycle, dispersal range, and survival rate were studied using one or combinations of these methods [13, 16, 18, 21–23]. Studies of host selection relied on indoor and outdoor RC to recover blood-fed mosquitoes [17, 23, 24], but this approach has serious limitations in the PNG setting. All of the species tend to be exophagic [25], thus few individuals (mostly human-fed ones) are found resting indoors, resulting in insufficient and biased samples. The wide dispersal range of some of these species along with thick tropical vegetation makes the outdoor resting search for mosquitoes in peri-domestic environments a laborious task, often resulting in very few mosquitoes that also do not adequately represent the population [23].

The barrier screen sampling (BSS) method was developed as an alternative to the methods discussed above [26–28]. It involves the use of agricultural shade cloth positioned vertically around villages and imposing a physical barrier suitable for temporary landing and resting of *Anopheles* mosquitoes as they commute into and out of the villages. Mosquito collectors visit the barrier screens at specific intervals throughout the night to capture the resting mosquitoes. Unlike the indoor resting collection, both anthropophilic and zoophilic mosquitoes may be intercepted as they rest on the barrier screen. By sampling the mosquitoes as they commute into and out of a village throughout the night, the BSS method overcomes the laborious task associated with outdoor resting collection. The flexibility in screen placement allows sampling in various locations which reduces bias associated with particular sampling locations. The effectiveness of this method to produce a sample of blood-fed mosquitoes for estimating host selection tendencies of several species of *Anopheles* vectors of malaria in PNG has been reported [28]. However, its application for studies of other behavioural or ecological aspects of *Anopheles* populations in PNG has not been reported. In this study, the BSS method was used to analyse species abundance, composition and nocturnal movement pattern of *Anopheles* species in the coastal and inland lowland malaria-endemic areas of PNG.

## Methods

### Study sites

**Fig. 1:**
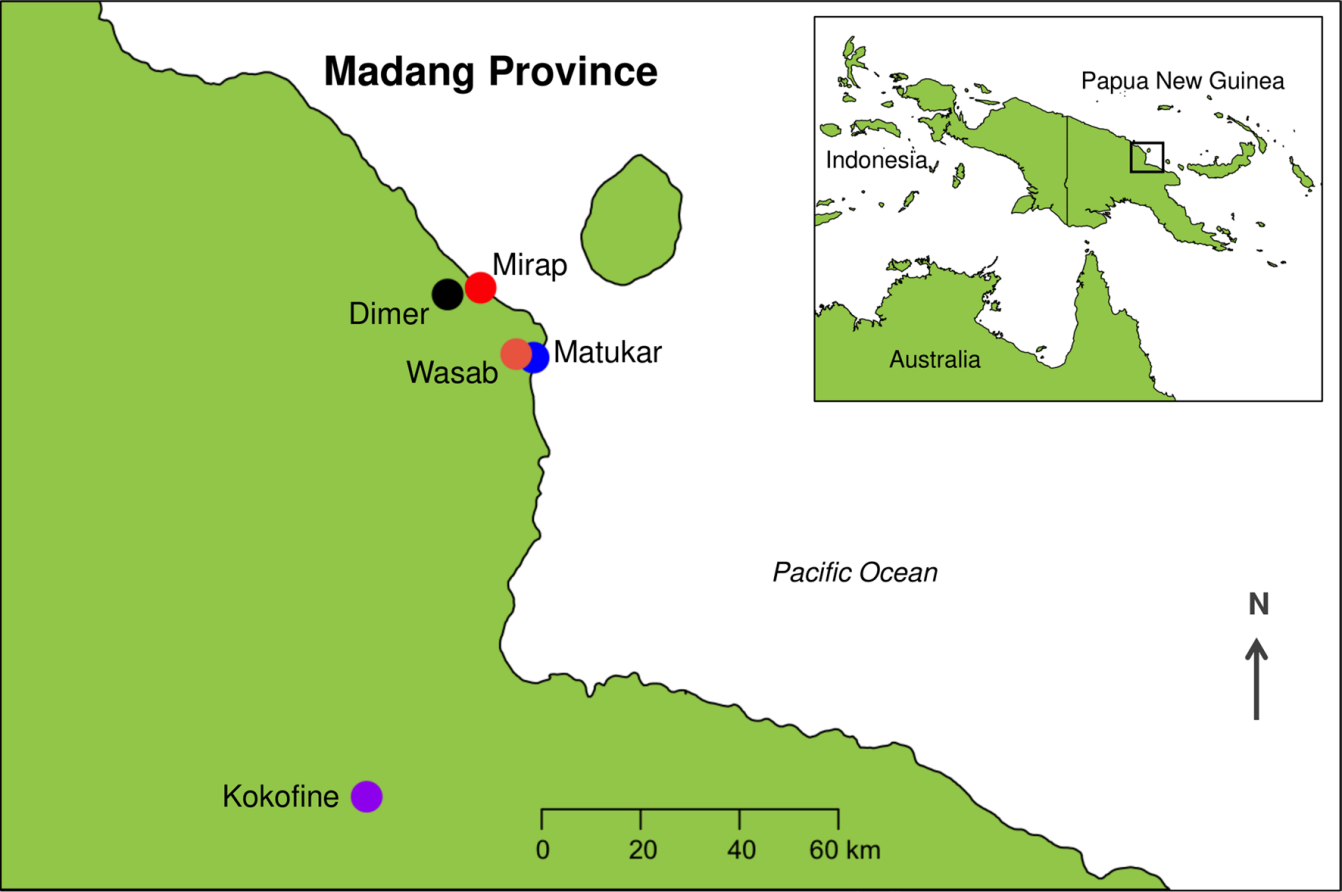
Map showing location of the study villages Dimer (black), Kokofine (purple), Matukar (blue), Mirap (red) and Wasab (orange) in Madang Province, Papua New Guinea. Areas shaded green represent landmasses

This study was conducted in five rural villages (Dimer, Kokofine, Matukar, Mirap and Wasab) in Madang province of PNG (Fig. 1) from 2012 to 2016. These villages are located in three ecogeographic environments of the malaria-endemic region of northern PNG (coastal plain, hilly inland terrain, and inland alluvial plain) and are inhabited by various *Anopheles* species, including the seven vector species listed above [13, 16–18, 23, 29–32]. The villages Matukar and Mirap share similar features; both are situated on coastal plain along the northern coastline just above sea level. Land cover consists of coconut plantation, secondary forest, vegetable gardens, brackish swamps, houses, foot trails, and exposed soil. The villages border sand beach and shore of the Pacific Ocean. Wasab and Dimer villages are situated several km inland from this coastline, on elevated hilltops about 150 m above sea level, with land cover and topography of steep-sided, forested hills and numerous streams flowing to rivers in nearby valleys. The fifth village, Kokofine, is situated on the alluvial plain of the Ramu river, 39 km from the coast and 400 m above sea level. Land cover there consists primarily of swamps, cocoa plantations and secondary forests. All villages have the same tropical climate condition of hot and wet with average atmospheric temperature of 28 °C.

### Mosquito sampling

The structure and set up of barrier screens were similar to those described elsewhere [26–28]. In this study, each barrier screen consisted of a 20 m long and 2 m wide polyethylene shade cloth (forest green, 70% shading grade) fastened on wooden poles or metal reinforcement bars and erected vertically to a height of 2.15 m. The length of the barrier screen was chosen for efficiency of setting up the screen and the amount of time spent searching the barrier screen surface for resting mosquitoes. The height of the barrier screen was consistent with the ability of the collectors to reach for mosquitoes just above their average height (160 cm). The barrier screens were placed between the village perimeter and surrounding environment or bush. Each barrier screen was positioned with one side of the screen facing the village, hereafter referred to as “village side”, and the other facing away from the village, hereafter referred to as “bush side”. The barrier screen intercepts host-seeking and blood-fed mosquitoes as they commute into and out of the village.

Mosquitoes were sampled using two or ten barrier screens per village per night (see Additional file 1: Table S1 for the number of barrier screens deployed in a particular night in each village). Each barrier screen was assigned two trained mosquito collectors. One collector worked from 6 pm to midnight and was replaced by the second collector who worked from midnight to 6 am. The collectors, who were stationed 20 m from the screen, visited both sides of the screen three times within each hour with approximately 20 min sampling interval, which involved 5 min commuting between the barrier screen and collector station, another 5 min searching and aspirating resting mosquitoes on the barrier screen, and 10 min break before next visit to the barrier screen. As this study was among the first to test the BSS method, no prior information was available to guide the sampling strategy for this study. Mosquitoes were believed to rest only temporarily on the barrier screen, thus searching the barrier screen three times per hour was intended to maximize the number of mosquitoes collected. The collector walked along the barrier screen and collected resting mosquitoes with the aid of a flashlight and a mouth aspirator. The collectors were provided with and instructed to apply mosquito repellents on their bodies to deter mosquitoes from biting them. Captured mosquitoes were placed into screened cups labelled with the hour of the night and the side of the barrier screen (i.e., bush or village side) on which the mosquitoes were captured. Information for each mosquito, including date and hour of collection, the screen side it rested on, and the blood meal status (blood-fed or unfed) were recorded. Mosquitoes were sampled for seven nights in Matukar, 58 in Mirap, 49 in Wasab, 12 in Dimer and six in Kokofine during the years 2012, 2013, 2015 and 2016 (see Additional file 1: Table S1). To minimize sampling biases associated with fixed locations, each barrier screen was changed to a new location on each night of sampling.

With the aid of a light microscope, mosquitoes were sorted by sex. Males which were very few (ca. 1% of the mosquito sample) were identified and discarded. Female *Anopheles* were identified to their morphological species [33, 34], assigned a unique serial number, and stored dry on silica gel desiccant. Mosquitoes morphologically identified as member of the *Anopheles punctulatus* sensu lato (*s.l*.) group were subjected to a PCR assay [35] to identify the species.

### Statistical analyses

Similarity in species composition between pairs of villages was estimated by performing Bray–Curtis index analysis [36] on mosquito abundance matrix for year 2012 only when mosquitoes were sampled in all five villages (Table 1). The resulting Bray–Curtis dissimilarity index matrix (Additional file 2: Table S2) was used in principal coordinates analysis (PCoA) to produce an ordination plot of the study villages. The plot was further modified as a biplot of villages and *Anopheles* species by a posteriori projection of the species onto the PCoA axes based on their weighted average scores. The weighted average score of a species at PCoA axis 1 and axis 2 was calculated by averaging the product of its abundance (Table 1) and the PCoA axis (1 or 2) score for all villages [37]. Villages clustered together in the biplot are similar whereas those further apart are dissimilar in their species composition. The position of a village in the PCoA biplot was influenced by the abundance of the *Anopheles* species closest to it [37]. The Bray–Curtis index matrix and PCoA biplot were computed using the functions *vegdist*, *cmdscale* and *wascores* of the package *vegan* [38] in R software (version 3.4.1). For each of eight *Anopheles* populations, generalized linear model (GLM) with negative binomial distribution was used to compare number of mosquitoes in collections at each of the 12 hourly periods of the night, from the village and bush side of the barrier screen, and by blood-fed and unfed status. In the GLM regression equation ln(*μ*) = *β*_0_ + *β*_1_(*Time*) + *β*_2_(*Side*) + *β*_3_(*Status*) + ln(*S*), the expected mosquito number *μ* was modelled as a linear function of the categorical predictor variables *Time*, with 12 levels representing the 12 hourly periods of the night; *Side*, with two levels representing the two sides of the barrier screens; and *Status*, with two levels representing blood-fed or unfed status of the mosquitoes. Number of barrier screen-hours *S* was included as the offset term. The GLM was performed using function *glm.nb* from R package *MASS* [39]. The proportion of blood-fed relative to unfed mosquitoes on the bush side of the barrier screen was compared to that of the village side using Chi square test of equality of proportions for each *Anopheles* population. Significance level for all statistical tests was based on type I error rate of 5%.

**Table 1:** Number of each *Anopheles* species (excluding *An. hinesorum*) collected in each of the five study villages in the year 2012 only

Species	Dimer	Kokofine	Matukar	Mirap	Wasab
*An. bancroftiii*	11	0	0	127	3
*An. farauti s.s.*	6	0	141	1775	4
*An. farauti* no. 4	0	1520	0	0	0
*An. koliensis*	9	0	2	11	13
*An. longirostris*	16	0	28	45	275
*An. punctulatus s.s.*	179	0	19	60	147

## Results

### Species composition and abundance

**Fig. 2:**
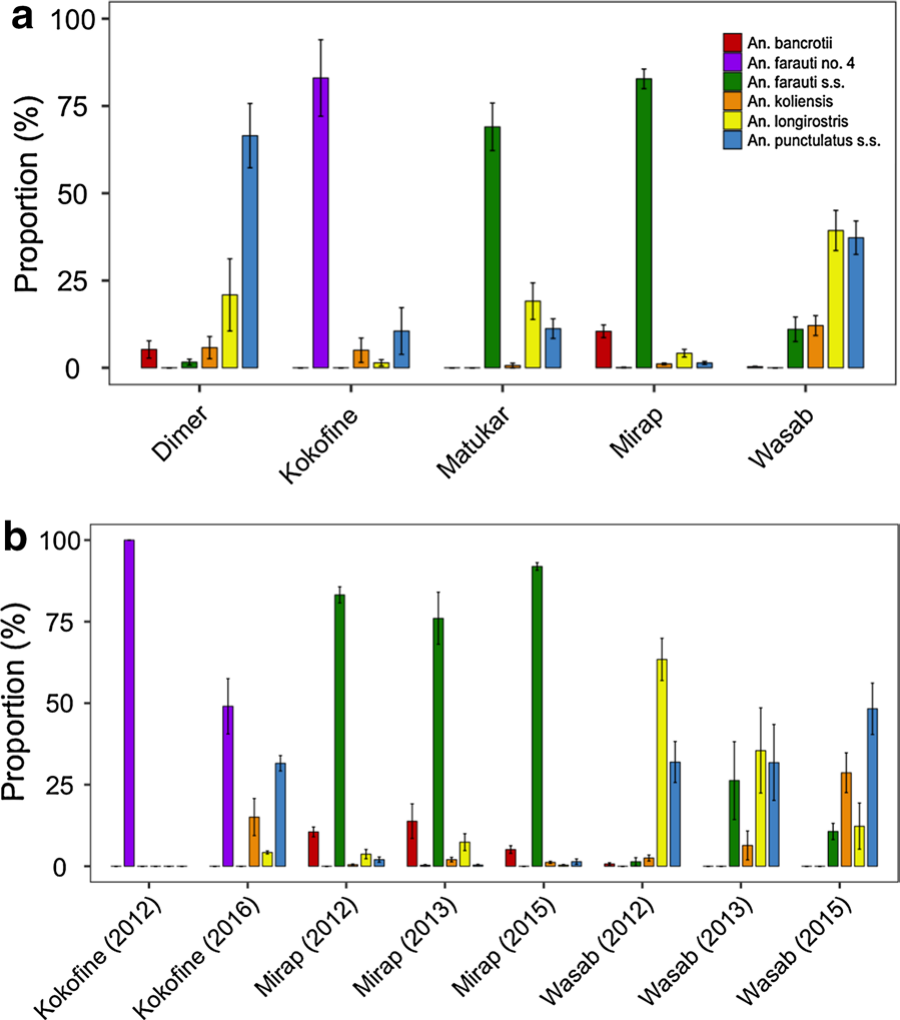
**a** Mean proportion (± se) of each *Anopheles* species captured on barrier screens each night in each village. The total number of mosquitoes (i.e. all sampling nights combined) captured in each village are Dimer (n = 221), Kokofine (n = 1732), Matukar (n = 193), Mirap (n = 3985) and Wasab (n = 1015). **b** Mean proportion (± se) of each *Anopheles* species captured on barrier screens each night in Kokofine in the years 2012 (n = 1520) and 2016 (n = 212); Mirap in 2012 (n = 2018), 2013 (n = 821) and 2015 (n = 1141); and Wasab in 2012 (n = 442), 2013 (n = 69) and 2015 (n = 504)

A total of 7146 female *Anopheles* mosquitoes of seven different species (*An. bancroftii*, *An. farauti s.s*., *An. farauti* no. 4, *An. hinesorum*, *An. koliensis*, *An. longirostris* and *An. punctulatus s.s*.) were collected, including 2611 (36.5%) blood-fed and 4535 (63.5%) unfed; gravid females were very low in numbers (< 0.1%, and not considered further). The distribution of mosquito numbers for each of the *Anopheles* species over the five study villages is shown in Table 2. The mean proportion (± se) of each *Anopheles* species (excluding *An. hinesorum*, due to low sample size) collected per barrier screen per night in each village is shown in Fig. 2a. The results presented in Tables 1 and 2 and Fig. 2a show that these *Anopheles* species were not evenly represented within and among villages. In Matukar, where five different species were found, *An. farauti s.s.* constituted 69.0 ± 7% of the mosquitoes sampled per barrier screen per night (Fig. 2a). In Mirap, where six species were found, *An. farauti s.s.* constituted 82.8 ± 3% of the sample (Fig. 2a). In Kokofine, where four species were found, *An. farauti* no. 4 predominated (83.0 ± 11%) the sample and in Dimer, where five species were found, *An. punctulatus s.s.* predominated (66.5 ± 9%) the sample (Fig. 2a). In Wasab, where five species were found, *An. longirostris* (39.3 ± 6%) and *An. punctulatus s.s.* (37.3 ± 5%) each constituted a similar, high proportion of the sample while the rest of the species were relatively less abundant (Fig. 2a).

**Table 2:** Number of each *Anopheles* species collected in each of the five study villages in the years 2012–2016

Species	Dimer	Kokofine	Matukar	Mirap	Wasab
*An. bancroftiii*	11	0	0	223	3
*An. farauti s.s.*	6	0	141	3557	78
*An. farauti* no. 4	0	1627	0	0	0
*An. hinesorum*	0	0	0	8	0
*An. koliensis*	9	32	2	39	209
*An. longirostris*	16	9	28	77	340
*An. punctulatus s.s*.	179	67	19	81	385

For Kokofine, Mirap and Wasab where mosquitoes were sampled for more than 1 year, variation in species composition between the years was observed (Fig. 2b). In Kokofine, the species composition changed from solely *An. farauti* no. 4 in 2012 to a more diverse community of four species in 2016. The proportions of these four species in 2012 and 2016 was significantly different (general Chi square test: χ^2^ = 825.8, df = 3, P < 0.0001). In Wasab, *An. farauti s.s., An. koliensis* and *An. punctulatus s.s.* increased in proportion whereas *An. longirostris* decreased from 2012 to 2015 and this change was statistically significant for five *Anopheles* species (*An. farauti* no. 4 was excluded due to zero data in all 3 years) (χ^2^ = 463.0, df = 8, P < 0.0001). In contrast, although the proportions of the five *Anopheles* species (*An. farauti* no. 4 was excluded due to zero data) in Mirap differed statistically (χ^2^ = 66.8, df = 8, P < 0.0001), between the 3 years, this change in species proportion was not visually dramatic as in Wasab and Kokofine because the change happened only in the less abundant vector species but the proportion of the primary vector *An. farauti s.s*. remained generally steady over the 3 years (Fig. 2b). Comparison of mosquito abundance among the villages based on data from the year 2012, when mosquitoes were sampled in all five villages (see Additional file 1: Table S1), showed great variation in the number of *Anopheles* mosquitoes (regardless of species) captured per barrier screen per night among the villages. Kokofine had the highest abundance with an average of 170 *Anopheles* per barrier screen per night followed by Mirap (36 per night), Matukar (13 per night), Wasab (11 per night) and Dimer (10 per night) in decreasing order.

**Fig. 3:**
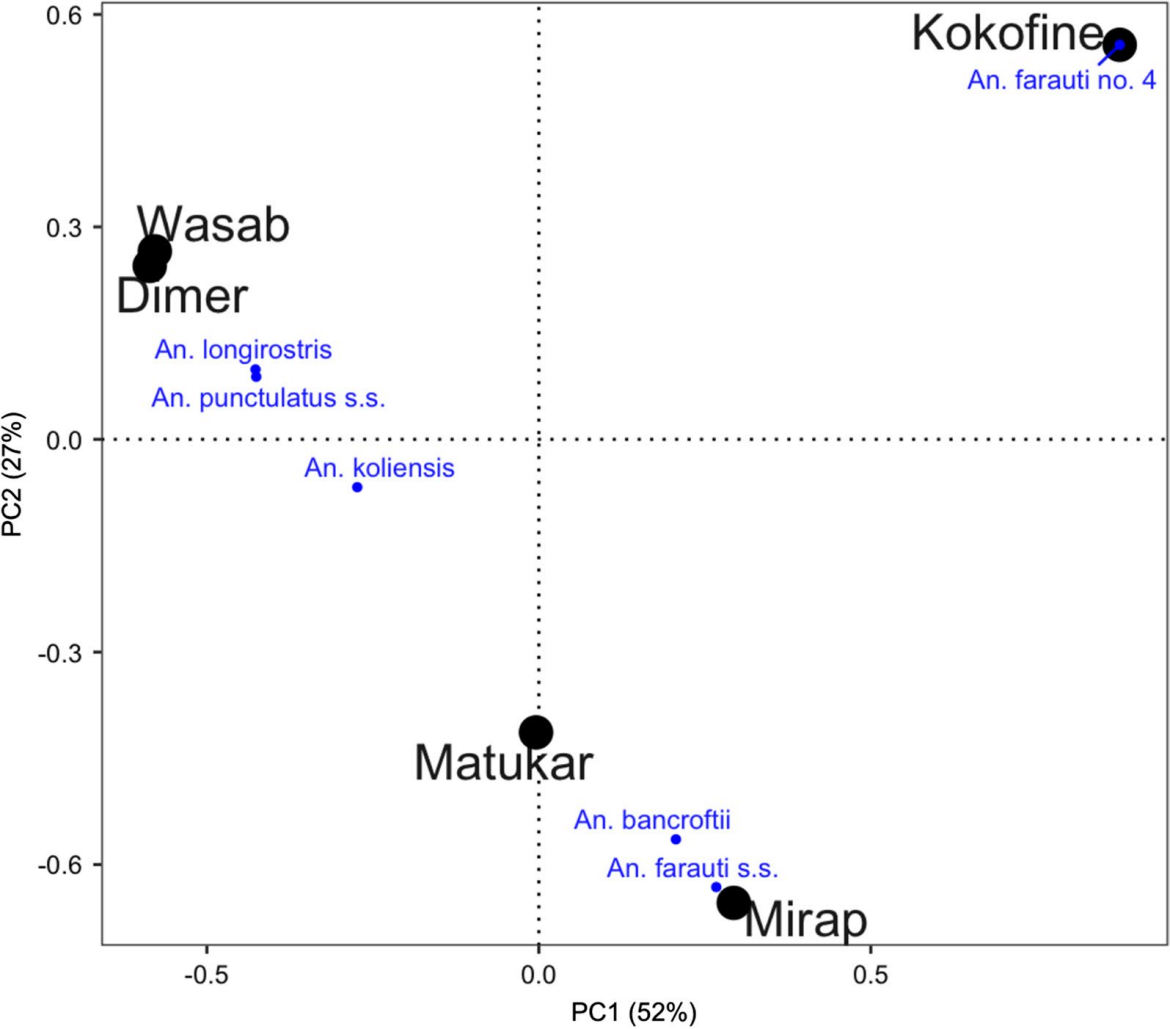
PCoA biplot based on Bray–Curtis index matrix of five villages (black) with projected weighted average scores of six *Anopheles* species (blue). The importance of the two orthogonal axes labelled PC1 and PC2 was determined by their eigenvalues and the percentage represents the proportion of variation explained by each axis. The species *An. hinesorum* was excluded from the analysis due to very low sample size

Ordination of the villages based on the Bray–Curtis dissimilarity matrix (Additional file 2: Table S2) showed that Dimer and Wasab closely clustered together in the PCoA biplot; Matukar and Mirap also clustered together but less closely; and Kokofine was separate from the other villages (Fig. 3). The close grouping of the two inland villages Dimer and Wasab was influenced by the similar presence and abundance of *An. koliensis*, *An. punctulatus s.s.* and *An. longirostris* in the year 2012 (other years were excluded from the analysis due to lack of sampling in some villages in those years). Similarly, the grouping of the two coastal villages Matukar and Mirap was influenced by the similar abundance and dominance of *An. farauti s.s*. in both villages. However, their cluster was not as tight as the two inland villages because of *An. bancroftii*, which was present and abundant in Mirap, but absent in Matukar. Kokofine differed greatly from the other villages, owing to the presence of *An. farauti* no. 4, which was the most abundant and exclusively found in that village (Fig. 3).

### Comparison of mosquitoes on bush and village side of barrier screen

**Fig. 4:**
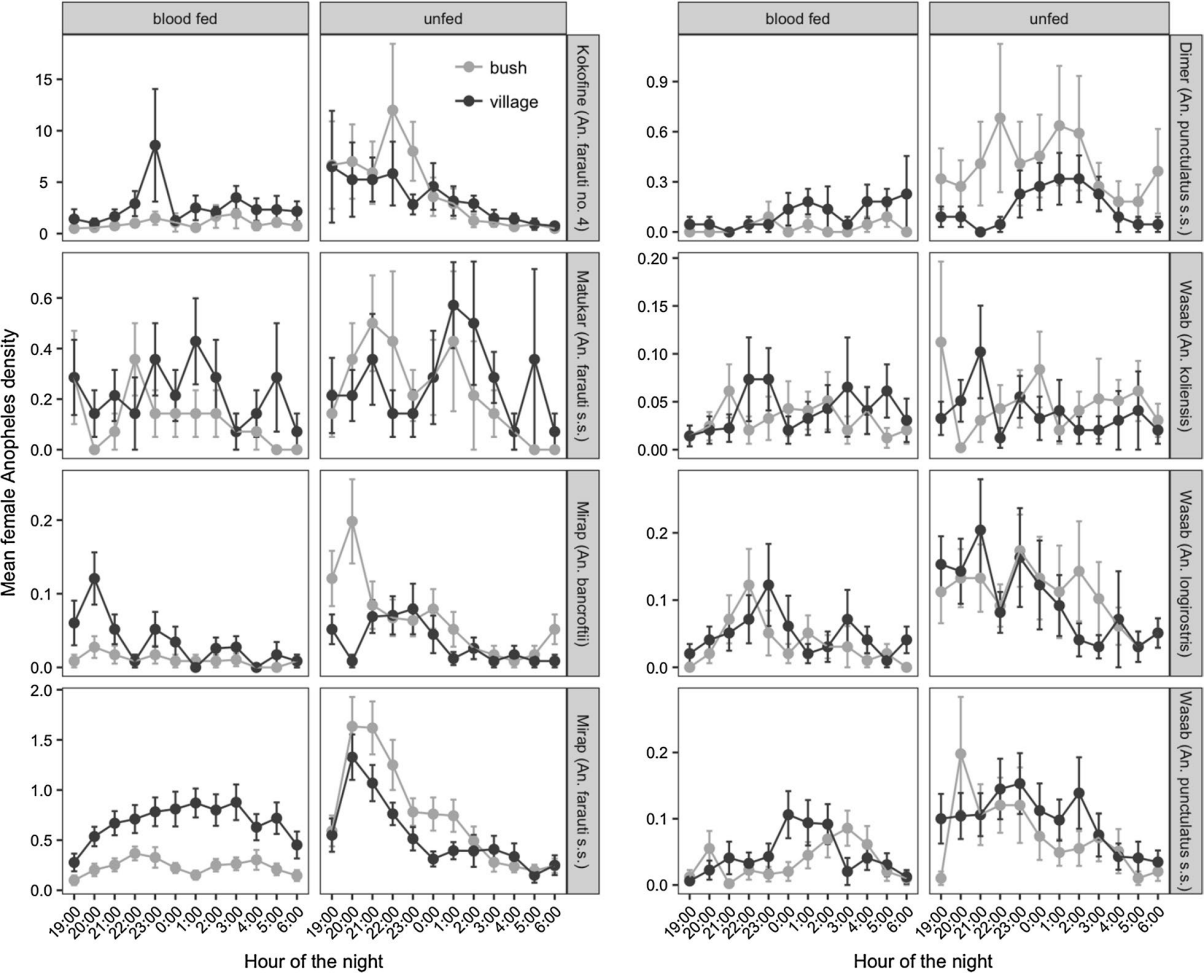
Mean number (± se) of blood-fed and unfed mosquitoes caught per barrier screen per night on the bush and village side of the screen (y-axis) at each of the 12 h periods of the night (x-axis) for eight *Anopheles* populations

**Fig. 5:**
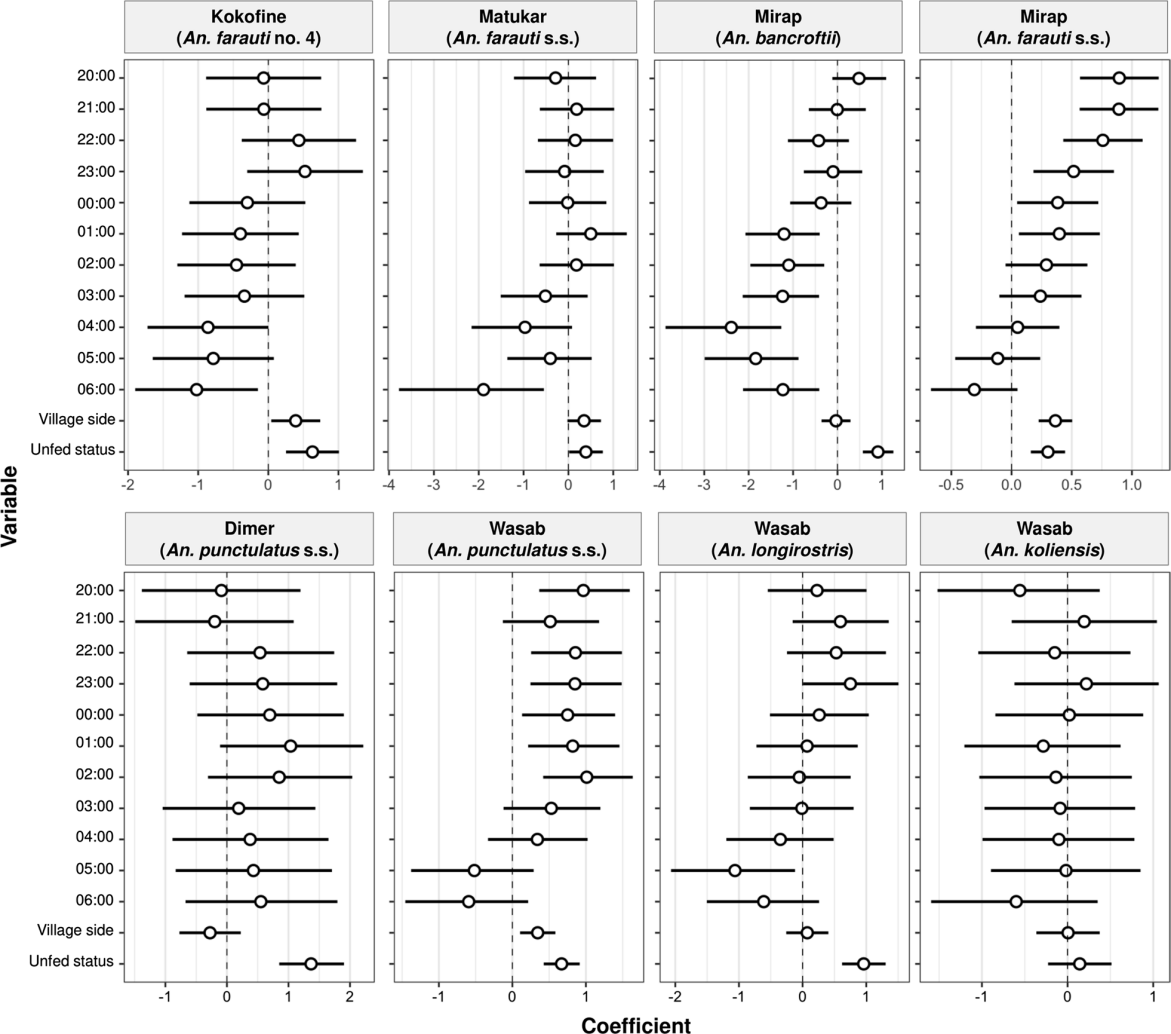
A plot of GLM estimated coefficients (open circle) with 95% confidence interval bars of 13 covariate levels (or variables) for eight *Anopheles* populations. The covariate levels plotted on the y axis include eleven of the twelve hourly periods of the night (expressed in 24 h format), village side of the barrier screen and unfed status. Each of the eleven hourly periods was compared with 19:00 h (not plotted) as the reference covariate level; the village side of the screen was compared with the bush side (not plotted) as the reference; and the unfed status was compared with fed status (not plotted) as the reference. The coefficient estimates (x axis) are in logarithmic scale

**Fig. 6:**
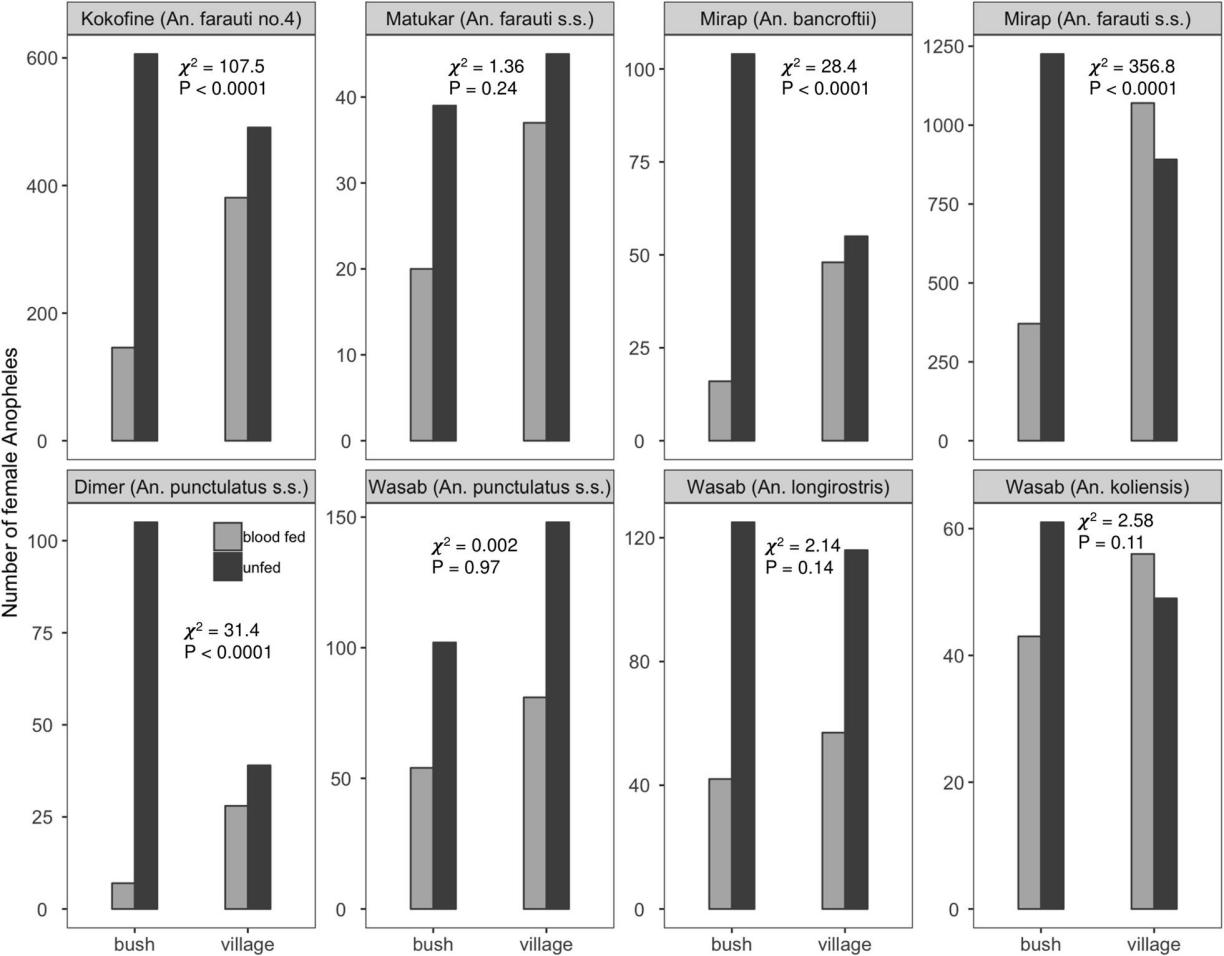
Barplot of blood-fed and unfed mosquito numbers captured on the bush and village side of the barrier screen for eight *Anopheles* populations. Chi square statistic and P values for test of equality of proportions for blood-fed relative to unfed mosquitoes on the bush and village sides of the barrier screen are shown within each panel. Degrees of freedom = 1 for all eight test categories

For selected species in each village (*An. farauti* no. 4 in Kokofine, *An. farauti* s.s. in Matukar, *An. bancroftii* and *An. farauti s.s*. in Mirap, *An. punctulatus s.s*. in Dimer, and *An. koliensis*, *An. longirostris* and *An. punctulatus s.s*. in Wasab), the mean number (± se) of blood-fed and unfed mosquitoes captured on the bush and village side per barrier screen per night were plotted for each of the 12 hourly periods of the night (Fig. 4). Full statistical output of the GLM analysis for these *Anopheles* populations are presented in Additional file 3: Table S3 and summarized graphically in Fig. 5. Except for *An. punctulatus s.s.* in Dimer and *An. koliensis* in Wasab which had a statistically similar number of mosquitoes collected throughout the night, the other six populations had statistically different numbers of mosquitoes for one or more of the hourly periods of the night compared to 19:00 h (Fig. 5, Additional file 3: Table S3). The GLM coefficient plot (Fig. 5) showed that except *An. punctulatus s.s*. in Dimer and *An. koliensis* in Wasab, the other six populations had higher number of mosquitoes in the hourly periods before midnight and less mosquitoes in the hours after midnight, after controlling for side of barrier screens and feeding status. This trend was strongly expressed in the unfed host-seeking subgroup of the six populations (Fig. 4; bush side, unfed panels). The number of mosquitoes captured on the village side of the barrier screens was significantly higher than the bush side for *An. farauti* no. 4 in Kokofine, *An. farauti s.s*. in Matukar, *An. farauti s.s*. in Mirap and *An. punctulatus s.s*. in Wasab, but not the other four populations (Fig. 5, Additional file 3: Table S3). For all populations except *An. koliensis* in Wasab, significantly more unfed than blood-fed mosquitoes were collected by the BSS method (Fig. 5, Additional file 3: Table S3). The total number of blood-fed and unfed mosquitoes captured on the village and bush side of the barrier screens for each of the eight *Anopheles* populations is shown in Fig. 6. Chi square tests for equality of proportions applied to the data in Fig. 6 showed that the proportion of blood-fed relative to unfed mosquitoes was significantly higher on the village than bush side of the barrier screen whereas the proportion of unfed relative to blood-fed was higher on the bush than village side for the populations in Kokofine, Mirap and Dimer but not those in Matukar and Wasab (Fig. 6).

## Discussion

The effectiveness of the BSS method to produce an adequate and unbiased sample of blood-fed mosquitoes for estimating host selection tendencies of mosquito vectors, and to study timing of host-seeking and infer flight behaviour, has been reported for several *Anopheles* species of the southwest Pacific, including PNG [26–28]. The *Anopheles* fauna encountered in our study villages using this method of sampling was limited to seven species, but species composition and dominance varied considerably, with certain species dominant in some villages (e.g., *An. farauti* no. 4 in Kokofine, *An. farauti s.s*. in Mirap) while completely absent (*An. farauti* no. 4) or present but relatively less dominant or common (*An. farauti s.s*. in Dimer and Wasab) in other villages. These findings are consistent with, and extend those, of two other studies using other sampling methods [13, 32]. In particular, the dominance of *An. farauti* s.s. in the coastal villages was reversed by greater relative abundance of *An*. *punctulatus* s.s. in the nearby inland villages (Fig. 2a). *Anopheles longirostris* was present across all sites at least in some years and typically a less common species, but was dominant in samples from Wasab for two of the 3 years (Fig. 2b).

The observed diversity of the species combined with variation in their ecological attributes [23, 24, 28, 40] can potentially attenuate single-intervention malaria vector control programmes such as long-lasting insecticide-treated bed nets (LLINs) in PNG. For example, a previous study showed that *An. koliensis* is highly anthropophilic whether or not LLINs were distributed in communities, whereas *An. longirostris*, *An. punctulatus s.s*., *An. farauti s.s.* and *An. farauti* no. 4, were more plastic in their host selection tendencies and diverted more feedings to pig and dog hosts when LLINs were in use [28]. Indeed, other studies have shown that *An. koliensis* populations greatly declined initially after an LLIN distribution campaign in PNG, whereas populations of *An. longirostris*, *An. punctulatus* s*.s*., *An. farauti s.s*. did not [13, 21]; a differential impact of LLIN that was likely due to the different host selection tendencies of the vector species. Thus, in villages like Wasab where vector diversity is high, and the species present utilize humans and other domestic vertebrate hosts for blood, the effectiveness of LLINs on malaria parasite transmission will be lessened. Such village level differences in species composition, even for those locations relatively close to each other, could explain the disparity in impact of vector control on malaria observed for a country-wide LLIN campaign in PNG [41]. Importantly, *An*. *koliensis* increased proportionately in the later years of the study period in Wasab and to a lesser extent Kokofine (Fig. 2b). This phenomenon suggests a decline in the control programme’s effectiveness, particularly reduced use of LLINs, but it could also be caused by seasonal variation, which was not captured in this study.

For five of the eight *Anopheles* populations analysed, the number of host-seeking mosquitoes (i.e., the bush side, unfed mosquitoes) arriving in the villages peaked between 8 pm and 10 pm in the evening and declined towards morning (Fig. 4). This relatively early arrival suggests that the adult resting sites and larval habitats for these populations were close to the villages, resulting in short commuting time between the habitats and the village. In contrast, the number of host-seeking mosquitoes in the other three populations (*An. punctulatus s.s*. in Dimer; *An. koliensis* and *An. longirostris* in Wasab) was extended across the evening, midnight, and morning hours (Fig. 4, unfed panels) suggesting that the adult resting sites for these mosquito populations are further from the village. This observation reflects an earlier study in the north coast villages of Madang which found that blood-fed individuals of *An. farauti s.l.* flew < 50 m from the study villages before resting in the nearby vegetation, whereas *An. punctulatus s.s*. and *An. koliensis* dispersed widely [23]. Those researchers attributed this variation in dispersal among the species to the proximity of their preferred larval habitats and resting sites to the villages. Similarly, in East Sepik province, biting rates of *An. farauti s.l*. and *An. longirostris* were highest in the evening and declined towards morning, whereas those of *An. koliensis* and *An. punctulatus s.s.* were lowest in the evening and peaked in the morning hours [32]. In Kokofine an early-evening biting pattern was observed for *An. farauti* no. 4 [18]. Generally, the primary host-seeking activity of most of these *Anopheles* populations coincides with evening activity of villagers who would, therefore, be unprotected by LLINs.

The higher proportion of blood-fed relative to unfed mosquitoes on the village than bush side of the barrier screen and higher proportion of unfed relative to blood-fed on the bush than village side of the screen is consistent with commuting behaviour of mosquitoes. For exophilic mosquito populations like those investigated here, freshly blood-fed mosquitoes must exit the village towards their resting sites in the surrounding vegetations and are therefore likely to be intercepted by the village than bush side of the barrier screen. Similarly, unfed host-seeking mosquitoes must enter the village from their resting sites to seek vertebrate hosts and are therefore likely to be intercepted by the bush than village side of the barrier screen.

This study has two important limitations. First, the presence of the mosquito collectors near the barrier screens during mosquito collections was an unavoidable aspect of the BSS method but it also introduced potential sampling bias in favor of anthropophilic vectors. To minimize this bias, the collectors applied insect repellents on their bodies and positioned themselves further from the barrier screen and among the inhabitants of the hamlets when they were not visiting the barrier screen. Results from blood meal analysis study [28] found that > 50% of blood-fed mosquitoes from a population of *An. farauti s.s.* and a population of *An. farauti* no. 4 fed on pigs or dogs. Mosquitoes in both of these populations are opportunistic feeders [28]. As these free-roaming nonhuman hosts did not visit the barrier screens like the human collectors, this result indicates minimal effect of the collector bias; a bias result would have shown significantly more human than nonhuman blood meals. Second, the times of the year during which mosquitoes were sampled within each village was not consistent over the years (see Additional file 1: Table S1). Also, mosquitoes were not collected at the same time in the different villages. As different species of *Anopheles* in PNG, particularly those within the *Anopheles* punctulatus group, exhibit affinity for specific larval habitat types and the temporal distribution and abundance of these habitat types are associated with annual rainfall pattern [40], these inconsistencies in sampling time may not capture any temporal or spatial pattern in the species composition and abundance associated with annual rainfall season. For example, *An. farauti* no. 4 whose bionomics is poorly understood but believed to be associated with riverine puddles formed along the flood plains of Ramu River after a flooding event, was the only species collected in June of 2012 in Kokofine, but species richness rose to four in February 2016. Although it is possible that this change in species composition in 2016 could have resulted from a major ecological change after 2012, it is likely the result of annual seasonal variation which was not controlled for in this study.

## Conclusion

This study shows that in PNG, *Anopheles* species abundance and composition varied greatly among sites, even those that are less than one km apart. Such local heterogeneity in species composition can complicate vector control efforts in PNG. For most of the *Anopheles* populations, the majority of the host-seeking mosquitoes arrived in the village before midnight when most people were active and exposed to the mosquitoes. In areas where *Anopheles* mosquitoes exhibit this temporal host-seeking behaviour, the LLIN programme will be ineffective against malaria. In addition to its effectiveness in sampling blood-fed mosquitoes for analysis of their host selection tendencies, the BSS method is useful for analysing other aspects of the vector populations. By serving as an interception device, the BSS system permits inferences about local movement patterns including nocturnal activity of mosquito populations. Finally, two potential limitations associated with this study were discussed: sampling bias in favor of anthropophilic mosquitoes due to presence of human collectors near the barrier screen and effect of seasonal patterns not accounted for in the temporal and spatial analyses of species composition and abundance.

## Additional files

**Additional file 1: Table S1.** Dates of mosquito sampling in each village in the years 2012, 2013, 2015 and 2016.

**Additional file 2: Table S2.** Bray–Curtis dissimilarity index matrix based on species abundance data for year 2012 only.

**Additional file 3: Table S3.** Statistical output for negative binomial generalized linear model analysis of eight *Anopheles* populations.

## Abbreviations

BSS: barrier screen sampling
HBR: human biting rate
HLC: human landing catch
IBR: infectious biting rate
LLINs: long-lasting insecticide-treated bed nets
LT: light trap
PCoA: principal coordinate analysis
PNG: Papua New Guinea
RC: resting collection
