# Supplementary material for: Species abundance, composition, and nocturnal activity of female Anopheles (Diptera: Culicidae) in malaria-endemic villages of Papua New Guinea: assessment with barrier screen sampling
Source: Malar J. 2019 Mar 25;18:96. doi: 10.1186/s12936-019-2742-x (PMC6434780; doi:10.1186/s12936-019-2742-x)
Supplement: Supplementary file 2 — Additional file 2: Table S2. Bray–Curtis dissimilarity index matrix based on species abundance data for year 2012 only. [file 12936_2019_2742_MOESM2_ESM.docx]

**Table S2**. Bray-Curtis dissimilarity index matrix based on species abundance data for year 2012 only. Values closer to 0 indicate villages with similar species composition and values closer to 1 indicate dissimilar villages.

|  | Dimer | Kokofine | Matukar | Mirap | Wasab |
| --- | --- | --- | --- | --- | --- |
| Dimer | 0.000 | - | - | - | - |
| Kokofine | 1.000 | 0.000 | - | - | - |
| Matukar | 0.791 | 1.000 | 0.000 | - | - |
| Mirap | 0.9088 | 1.000 | 0.828 | 0.000 | - |
| Wasab | 0.460 | 1.000 | 0.832 | 0.900 | 0.000 |
